# Spinal tuberculosis in Afghanistan: A 2019‐2020 study of patients in Kabul hospitals

**DOI:** 10.1002/puh2.120

**Published:** 2023-09-30

**Authors:** Farah Qaderi, Idriss Mashid, Latif Gachkar, Mosè Martellucci, Shohra Qaderi, Akihiko Ozaki, Alireza Haghbin Toutounchi, Afshin Taheriazam, Takanori Asakura, Hojat Gholipoor Talemi, Noria Mohammady, Minoosh Shabani

**Affiliations:** ^1^ School of Medicine Shahid Beheshti University of Medical Sciences Tehran Iran; ^2^ Department of Medicine Kabul University of Medical Sciences Kabul Afghanistan; ^3^ Department of Infectious Diseases and Tropical Medicine Loghman Hakim Hospital Shahid Beheshti University of Medical Sciences Tehran Iran; ^4^ Infectious Diseases and Tropical Medicine Research Center Shahid Beheshti University of Medical Sciences Tehran Iran; ^5^ Department of Medicine University of Perugia Perugia Italy; ^6^ Jyoban Hospital of Tokiwa Foundation Iwaki Fukushima Japan; ^7^ Department of Orthopedics Faculty of Medicine Tehran Medical Sciences Islamic Azad University Tehran Iran; ^8^ Division of Pulmonary Medicine Department of Medicine Keio University School of Medicine Shinjuku‐ku Tokyo Japan

**Keywords:** Afghanistan, back pain, endemic, spinal tuberculosis, tuberculosis

## Abstract

**Background:**

Tuberculosis (TB) is endemic in many low‐income countries, which can affect the spine in 1%–5% of those with an active infection. This study reports the clinical characteristics of patients admitted for spinal TB in Kabul, Afghanistan, a country with very limited resources.

**Methods:**

This was a descriptive study among 26 patients treated for spinal TB in three major referral hospitals in Kabul, Afghanistan, between March 2019 and April 2020. The sociodemographic and clinical details, gender, age, site of infection, presenting complaints, signs, and symptoms of the patients were retrieved from their medical records. Summary statistics were analyzed using SPSS version 20. Ethics approval was obtained from the ethics committee of Shahid Beheshti University of Medical Sciences in Tehran, Iran.

**Results:**

Data were available for 26 patients with spinal TB admitted consecutively. The mean age of the sample was 38.2 years (SD 17.5), and there were 16 males and 10 females. Median time from symptom onset to hospital admission was 60 days. The most common diagnostic imaging technique used was magnetic resonance imaging (92.3%), followed by computed tomography (7.7%). The majority of the lesions involved the lumbar spine (61.4%), followed by the thoracolumbar spine (30.8%). Back pain was the most common manifestation in 21 patients (80.8%), and varying degrees of neurological impairment were found in 16 (61.5%) patients. There were no deaths among the patients up to the discharge date.

**Conclusions:**

This study describes the clinical characteristics of spinal TB among patients in Kabul, Afghanistan. It is essential to strengthen preventive strategies and to improve health awareness about clinical features of spinal TB in patients with chronic back pain even in the absence of signs of TB.

## Graphical abstract

1 Only half of the patients had a past history of pulmonary TB.

2 The majority of the lesions involved the lumbar spine (61.4%), followed by the thoracolumbar spine (30.8%).

3 Back pain was the main presenting symptom, in 21 patients (80.8%), and varying degrees of neurological impairment were found in 16 (61.5%) patients.

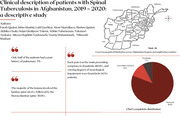

## INTRODUCTION

*Mycobacterium tuberculosis* is a common infectious agent, particularly in low‐income countries, with a reported incidence of 18.9 infection cases per 100,000 inhabitants each year in the general population of these, only 5%–15% become symptomatic, whereas the others remain latent [1, 2, 3]. The exact incidence of spinal tuberculosis (TB) has not been verified yet; however, extra‐pulmonary TB occurs in 20% of infected individual [4]. Out of these, skeletal TB is found in nearly 20% of patients, and the spine is the most common site of skeletal involvement (50% of patients with skeletal TB) [5].

Afghanistan is among one of the 22 high‐burden counties with TB with an incidence rate of 70,000 per year due to the World Health Organization (WHO) Global TB report, 2019 [1]. Unfortunately, the rate of mortality does not appear to be decreasing [6]. The southern part of Iran, where many Afghans live, reports an annual incidence of 70.3–80.6 per 100,000 population, including all types of TB and 13.8–19.4 per 100,000 population for extra‐pulmonary TB during 1997–2002 [6, 7]. TB is the second most frequent cause of Spinal Cord injury (SCI) in Afghanistan responsible for 16.3% of all SCI cases [8, 9]. In this study, the authors aimed to assess the clinical characteristics of patients admitted for spinal TB in three hospitals of Kabul, the capital city of Afghanistan.

## METHODS

### Study design, population, and setting

This was a descriptive study of spinal TB cases treated in the three major referral hospitals of Kabul (Shaikh Zayed Hospital, Ali Abad Hospital, and Jamhuriat Hospital). This study includes all patients with spinal TB cases admitted consecutively from March 2019 to April 2020.

### Data collection method

Spinal TB cases were defined as having culture for *M. tuberculosis* and/or compatible finding in microscopy tissues biopsy, computed tomography (CT) scan, magnetic resonance imaging (MRI).

The data were corroborated with clinical and demographic records, along with the latest recorded patient status. Documents were in the three languages used in Afghanistan (Farsi, Pashto, and English), which required a phase of translation prior to compiling the final database. All records were then evaluated by three licensed physicians for inclusion.

The measurements of the induration after purified protein derivative (PPD) skin tests, that is, Mantoux tests, were taken after 72 h. The follow‐up lasted until the last recorded clinical file, which could correspond to either the discharge date or the exits date.

### Data handling

We analyzed cases according to gender, age, site of infection and presenting complaints, signs, and/or symptoms. Summary statistics were mean and standard deviation (SD) or median and inter‐quartile range (IQR) for continuous variables, and number and percentages for categorical variables. Statistical significance was set as a two‐tailed *p* < 0.05. Data analysis was carried out with Statistical Package for the Social Sciences (SPSS) version 20.

### Ethical clearance

This study was given approval by the ethics committee of Shahid Beheshti University of Medical Sciences in Tehran, Iran, with registration code: (IR.SBMU.MSP.REC.1400.537). *The letter of permission from Shahid Beheshti University was further confirmed and authorized by the Afghan Embassy, and access to the file was granted by Kabul Medical University*.

## RESULTS

**FIGURE 1:**
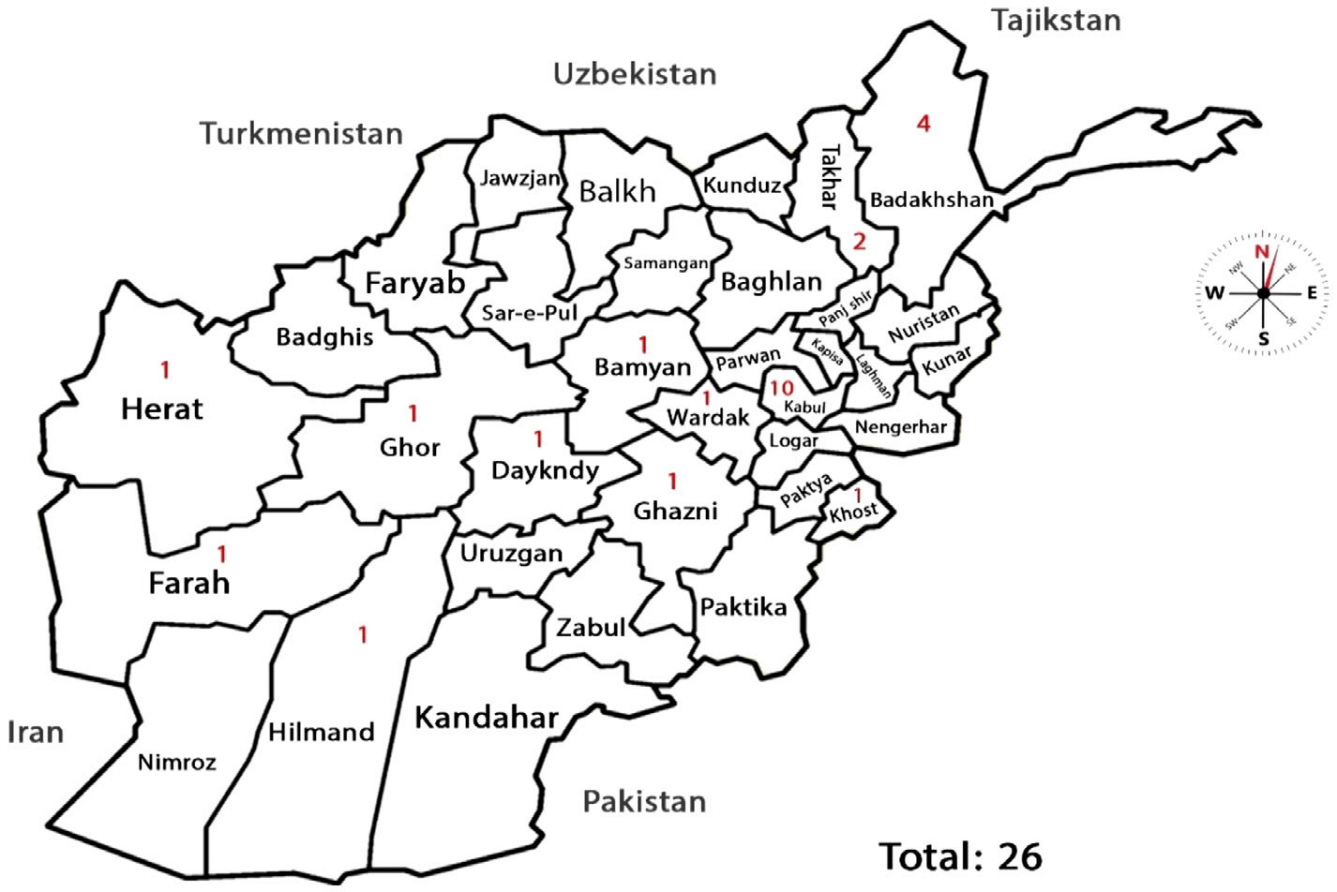
Geographical distribution of spinal tuberculosis cases from across Afghanistan, 2019–2020.

Between March 2019 and April 2020, 31 patients with a diagnosis of spinal TB were admitted to the three teaching hospitals. Five cases were excluded, due to insufficient data (three cases), multifocal bone involvement (one case), and malignancy (two cases). Overall, 26 cases were included in the study: 20 cases from Shaikh Zayed Hospital, 4 from Ali Abad Hospital, and 2 from Jamhuriat Hospital. Ten patients were from Kabul (38.5%), four from Badakhshan (15.4%), two from Takhar (7.7%), and the rest are shown comprehensively in Figure 1.

The mean age was 38.2 years (SD 17.5) and 16 (61.5%) were males. The most represented occupation among females was housewife (10 females), 6 males were construction workers, and 3 other males were school students (30%).

**FIGURE 2:**
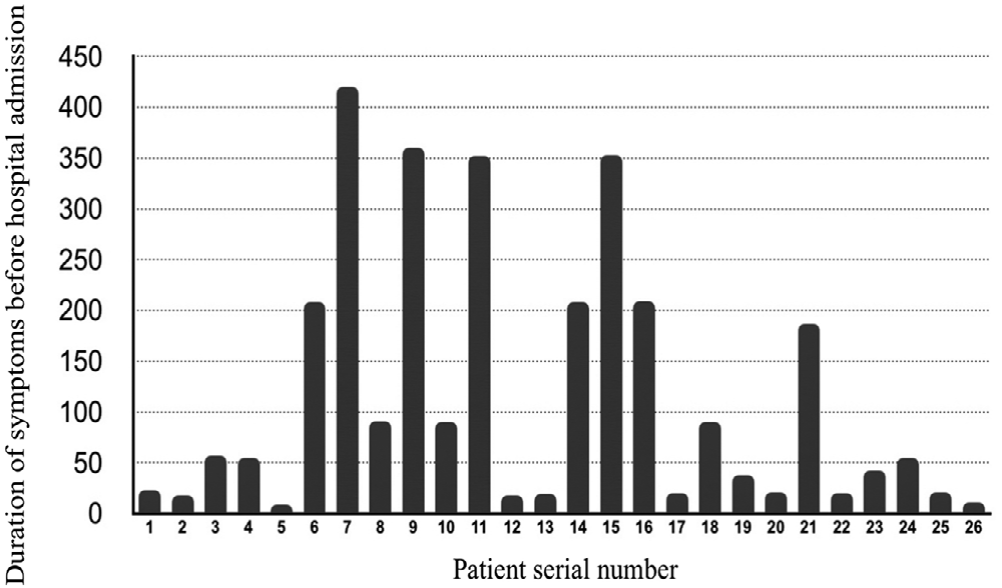
Time between symptoms onset and hospitalization of 26 patients with spinal tuberculosis in Afghanistan, 2019–2020.

The median time between symptoms onset and admission was 60 days, ranging from 10 to 420 days (Figure 2), whereas the median time of hospitalization was 6.5 days. About 40% of patients underwent surgery, whereas 60% were on medical treatment. Four of the patients (15.4%) were followed for 3 months, the time during which they recovered, whereas two patients refused treatment. There were no deaths among the patients up to the discharge date.

Approximately half of the patients had a past history of pulmonary TB (14 cases, 53.8%), whereas spinal TB was rarer (2 cases, 7.7%) and only 1 had polio infection, affecting the lower limb. The past history of TB treatment was not mentioned in medical records. None of the patients were positive for human immunodeficiency virus (HIV) or hepatitis B virus (HBV), and none had previously received the *Bacille Calmette‐Guérin* (BCG) vaccine.

### Clinical symptoms and signs

The leading cause of access to the hospitals for the included cases was back pain, reported by 21 (80.8%), while lower limb weakness was reported by 38.4% of patients (Table 1). Neurological deficits, weight loss and chronic cough were encountered in 61.5%, 73%, and 26.9% of patients, respectively (Table 2).

**TABLE 1:** Chief complaints/symptoms at presentation among patients with spinal tuberculosis in Afghanistan, 2019–2020 (*n* = 26).

Clinical characteristics	*n* (%)
*Chief complaint*	
Back pain	21 (80.8)
Lower limbs weakness	3 (11.5)
Paraplegia	2 (7.7)
*Associated symptom and/or signs*	
Weight loss^a^	19 (73.1)
Neurologic deficits	16 (61.5)
Lower limbs weakness	10 (38.4)
Chronic cough^b^	7 (26.9)
Urinary retention	5 (19.2)
Urinary incontinence	2 (7.7)
Loss of appetite	2 (7.7)
Kyphosis	1 (3.8)

^a^ >5% of body weight, over 6–12 months.

^b^ >8 weeks.

**TABLE 2:** Diagnostic imaging and tuberculin sensitivity findings among patients with spinal tuberculosis, Afghanistan, 2019–2020 (*n* = 26).

Imaging and laboratory findings	*n* (%)
*Magnetic resonance imaging*	24 (92.3)
Abscess	10 (38.5)
Discitis	10 (38.5)
Osteitis	7 (26.9)
Myelitis	3 (11.5)
Vertebral fracture	3 (11.5)
Disc destruction	7 (26.9)
≥1 of the findings above	24 (92.3)
≥2 of the findings above	12 (46.2)
≥3 of the findings above	2 (7.7)
≥4 of the findings above	1 (3.8)
*CT scan*	10 (38.4)
Vertebral fracture	2 (7.7)
Disc destruction	7 (26.9)
*PPD test (mm)*	
Median (Range)	16 (10–24)

### Radiological findings

The distributions of lesions along the spinal vertebrae were cervical in 1 patient (3.8%), thoracolumbar in 8 patients (30.8%), and lumbar in 17 (61.4%). Ten patients developed an abscess (38.5%), four of which were in the lumbar tract (15.4%), and three developed myelitis (11.5%). Almost half of the sample (46.2%) had at least two radiologic findings, including abscesses, discitis, osteitis, disc destructions, myelitis, or vertebral fracture. Only one patient presented with none of the previous manifestations. The MRI and CT imaging features are summarized in Table 2.

### PPD tests

Patients were tested for tuberculin reaction: after 72 h, the induration result ranged from 10 to 24 mm (median 16 mm, IQR 15.0–18.5—Table 2).

## DISCUSSION

Afghanistan is among the world's highly endemic region for TB in the world. Despite free medicines and diagnostics facilities in the county, TB continues to be a growing burden and a major health concern [10, 11]. According to the WHO report that 392,272 presumptive cases of TB had been tested for TB in 2017, in which 41% were bacteriologically confirmed, 27.5% were clinically diagnosed, 26% were extra pulmonary cases, and 20.5% of the cases were children [10]. Based on a single‐center study in Kabul, from a total of 118 cases, 14 cases (11.9%) were developed skeletal TB [12]. We found a considerable variation in the age at presentation, with approximately half of the sample between the ages of 20 and 50 years. Most patients were male, and from Kabul, whereas smaller proportions came from farther provinces of the country [13]. Importantly, the median delay in healthcare‐seeking from the onset of symptoms was long, at 2 months. This delay may be due to low health literacy, social stigma of having TB, getting isolated for a long term, and long distances from health‐care centers [14, 15].

Hospitalizations, conversely, were rather short, lasting around 1 week. There were no deaths registered in the study sample, and only a small part of them refused the treatment. The most frequent complaint was back pain, and virtually all patients had radiological findings indicating a severe involvement of the spine, with about one half presenting two or more severe TB manifestations such as abscesses or discitis that are fairly the same as the previous studies [16, 17]. Health‐care workers in Afghanistan should be aware of spinal TB in patients with complaining of localized back pain. It is crucial for health‐care providers in endemic region such as Afghanistan, to consider TB for the majority of Afghan population even with minimum to none signs of TB in order to prompt diagnosis.

In Zahedan, a province of Iran, due to the high awareness of physicians, for all Afghan nationals who visit Spinal Injury Ward, TB is checked even if they have no signs or symptoms of TB [6, 12]. One study that investigated extrapulmonary TB in Afghanistan up to 2008 found a greater proportion of women among the cases studied and outlined that 11.9% of the people included had skeletal TB, in which a male‐to‐female skeletal involvement of TB was 4:3, near to the findings in our study of 8:5 [12]. This result could be due to low vitamin D among Afghan females [18, 19, 20, 21, 22].

Afghanistan has a high burden of internally displaced people (particularly increased recently), poverty, living in crowded conditions, undernutrition, low level of education, and vitamin D deficiency that are risk factors for increasing TB burden in the country [18, 23–27]. To prevent TB‐related morbidity and mortality, it is important to strengthen the health system and encourage more people to seek help for this condition.

Our study has limitations, the first being the short follow‐up with the difficulty in assessing post‐discharge outcomes. The representativeness of the sample may be limited, as the people presenting to the three Kabul hospitals may belong to a higher socioeconomic strata. Future studies should continue to monitor the numbers and conditions of spinal TB patients, and wider rigorous assessments of the burden of TB should be performed, in order to prioritize public health strategies for preventing infections and to promote early health‐care‐seeking behaviors among the citizens.

## CONCLUSIONS

This study provides important information on clinical characteristics of spinal TB in patients in Kabul. The findings of this study should contribute to improve physicians’ awareness to consider spinal TB in patients with chronic back pain. There is a need to strengthen health system, promote early health seeking, and provide appropriate care to patients with spinal TB in Afghanistan.

## AUTHOR CONTRIBUTIONS

Farah Qaderi, Idriss Mashid, Mosè Martellucci, and Shohra Qaderi conceived and designed the study and wrote the manuscript; Idriss Mashid and Hojat Gholipoor Talemi helped collect data; Shohra Qaderi, Alireza Haghbin Toutounchi, and Mosè Martellucci performed the statistical analysis and wrote the manuscript; Latif Gachkar, Minoosh Shabani, and Noria Mohammady confirmed the eligibility of the participants for the study and wrote the manuscript; Akihiko Ozaki, Afshin Taheriazam, Minoosh Shabani, and Takanori Asakura supervised the whole study and approved the final version of the manuscript.

## CONFLICT OF INTEREST STATEMENT

Shohra Qaderi is a Member of the Youth Editorial Board. To minimize bias, she has been excluded from all editorial decision‐making related to the acceptance of this article for publication.

## ETHICS STATEMENT

The study was ethically approved by the Institutional Review Board of Shahid Beheshti University of Medical Sciences in Tehran, Iran, with registration code: (IR.SBMU.MSP.REC.1400.537). No potentially identifiable human images or data are presented in this study.

## Data Availability

All data used in this study are available upon reasonable request from the corresponding author.

## References

[1] World Health Organization . Global Tuberculosis Report 2019 . World Health Organization; 2023. https://www.who.int/publications/i/item/9789241565714

[2] World Health Organization . Global Tuberculosis Report 2018. World Health Organization; 2018.

[3] Singh N , Paterson DL . *Mycobacterium tuberculosis* infection in solid‐organ transplant recipients: impact and implications for management. Clin Infect Dis. 1998;27(5):1266‐1277. 9827281 10.1086/514993

[4] Schirmer P , Renault CA , Holodniy M . Is spinal tuberculosis contagious? Int J Infect Dis. 2010;14(8):e659‐e666. 20181507 10.1016/j.ijid.2009.11.009

[5] Esteves S , Catarino I , Lopes D , Sousa C . Spinal tuberculosis: rethinking an old disease. J Spine. 2017;6(1):358.

[6] Moghtaderi A , R Alavi‐Naini V Rahimi‐Movaghar . Tuberculous myelopathy: current aspects of neurologic sequels in the southeast of Iran. Acta Neurol Scand. 2006;113(4):267‐272. 16542167 10.1111/j.1600-0404.2005.00576.x

[7] Ayazi K , Samsami M , Qaderi S , Farsad SM , et al. Spontaneous perforation as a fatal presentation of esophageal tuberculosis: a case report. Int J Surg Case Rep. 2021;78:197‐200. 33360335 10.1016/j.ijscr.2020.12.042 PMC7771039

[8] Michael M , K Roth . Against all odds: a qualitative study of rehabilitation of persons with spinal cord injury in Afghanistan. Spinal Cord. 2012;50(12):864‐868. 23032605 10.1038/sc.2012.113

[9] Eslami V , V Rahimi‐Movaghar . Early diagnosis of tuberculosis in Afghanistan is the best available strategy to prevent spinal cord injury. Spinal Cord. 2013;51(4):343‐343. 23208543 10.1038/sc.2012.156

[10] World Health Organization Afghanistan, Situation Update . World Health Organizatio; 2022. http://www.emro.who.int/afg/programmes/stop‐tuberculosis‐stb.html

[11] Khan IM , U Laaser . Burden of tuberculosis in Afghanistan: update on a war‐stricken country. Croat Med J. 2002;43(2):245‐247. 11885055

[12] Fader T , Parks J , Khan NU , Manning R , Stokes S , Nasir NA . Extrapulmonary tuberculosis in Kabul, Afghanistan: a hospital‐based retrospective review. Int J Infect Dis. 2010;14(2):e102‐e110. 19541522 10.1016/j.ijid.2009.03.023

[13] Qaderi F , Qaderi F , Tarki FE , et al. Generalized, non‐neonatial tetanus is a highly fatal disease in Afghanistan: a case series study. Int J Infect Dis. 2021;103:568‐572. 33340666 10.1016/j.ijid.2020.12.019

[14] Johansson E , Long NH , Diwan VK , Winkvist A . Gender and tuberculosis control: perspectives on health seeking behaviour among men and women in Vietnam. Health Policy. 2000;52(1):33‐51. 10899643 10.1016/s0168-8510(00)00062-2

[15] Long NH , Johansson E , Diwan VK , Winkvist A . Fear and social isolation as consequences of tuberculosis in VietNam: a gender analysis. Health Policy. 2001;58(1):69‐81. 11518602 10.1016/s0168-8510(01)00143-9

[16] Alothman A , Memish ZA , Awada A , et al. Tuberculous spondylitis: analysis of 69 cases from Saudi Arabia. Spine. 2001;26(24):E565‐E570. 11740373 10.1097/00007632-200112150-00020

[17] Walker A . A 15‐year assessment of controlled trials of the management of tuberculosis of the spine in Korea and Hong Kong: thirteenth report of the Medical Research Council Working Party on Tuberculosis of the Spine. J Bone Jt Surg. British volume. 1998;80(3). 10.1302/0301-620x.80b3.8544 9619936

[18] Azizi S , TM Tariq . Vitamin D deficiency among Afghan adolescents in Kabul. J Coll Physicians Surg Pak. 2019;29(11):1072‐1077. 31659965 10.29271/jcpsp.2019.11.1072

[19] Sita‐Lumsden A , Lapthorn G , Swaminathan R , Milburn HJ . Reactivation of tuberculosis and vitamin D deficiency: the contribution of diet and exposure to sunlight. Thorax. 2007;62(11):1003‐1007. 17526677 10.1136/thx.2006.070060 PMC2117124

[20] Gibney KB , MacGregor L , Leder K , et al. Vitamin D deficiency is associated with tuberculosis and latent tuberculosis infection in immigrants from sub‐Saharan Africa. Clin Infect Dis. 2008;46(3):443‐446. 18173355 10.1086/525268

[21] Wilkinson RJ , Llewelyn M , Toossi Z , et al. Influence of vitamin D deficiency and vitamin D receptor polymorphisms on tuberculosis among Gujarati Asians in west London: a case–control study. Lancet North Am Ed. 2000;355(9204):618‐621. 10.1016/S0140-6736(99)02301-6 10696983

[22] Nnoaham KE , A Clarke . Low serum vitamin D levels and tuberculosis: a systematic review and meta‐analysis. Int J Epidemiol. 2008;37(1):113‐119. 18245055 10.1093/ije/dym247

[23] Nhlema B , Kemp J , Steenbergen, G , Theobald S , Tang S , Squire SB . A systematic analysis of TB and poverty. WHO, Geneva, Switzerland (2003) 4 pp.

[24] Antunes JLF , EA Waldman . The impact of AIDS, immigration and housing overcrowding on tuberculosis deaths in São Paulo, Brazil, 1994–1998. Soc Sci Med. 2001;52(7):1071‐1080. 11266050 10.1016/s0277-9536(00)00214-8

[25] Rieder, HL . Epidemiologic Basis of Tuberculosis Control. International Union Against Tuberculosis and Lung Disease Paris; 1999.

[26] Wandwalo E , O Mørkve . Delay in tuberculosis case‐finding and treatment in Mwanza, Tanzania. Int J Tuberc Lung Dis. 2000;4(2):133‐138. 10694091

[27] Acuti Martellucci C , Qaderi S , Tanimoto T , Ozaki A . Afghan women and children's health: three main challenges under Taliban and COVID‐19. J Glob Health. 2021;11:03126. 34956634 10.7189/jogh.11.03126 PMC8684792

